# A novel cholinergic projection from the lateral parabrachial nucleus and its role in methamphetamine-primed conditioned place preference

**DOI:** 10.1093/braincomms/fcac219

**Published:** 2022-08-30

**Authors:** Teng He, Wenwen Chen, Yu Fan, Xing Xu, Hao Guo, Nanqin Li, Xue Lu, Feifei Ge, Xiaowei Guan

**Affiliations:** Department of Human Anatomy and Histoembryology, Nanjing University of Chinese Medicine, Nanjing 210023, China; Department of Human Anatomy and Histoembryology, Nanjing University of Chinese Medicine, Nanjing 210023, China; Department of Human Anatomy and Histoembryology, Nanjing University of Chinese Medicine, Nanjing 210023, China; Department of Physiology, College of Korean Medicine, Daegu Haany University, Daegu 42158, South Korea; Department of Human Anatomy and Histoembryology, Nanjing University of Chinese Medicine, Nanjing 210023, China; Department of Human Anatomy and Histoembryology, Nanjing University of Chinese Medicine, Nanjing 210023, China; Department of Human Anatomy and Histoembryology, Nanjing University of Chinese Medicine, Nanjing 210023, China; Department of Human Anatomy and Histoembryology, Nanjing University of Chinese Medicine, Nanjing 210023, China; Department of Human Anatomy and Histoembryology, Nanjing University of Chinese Medicine, Nanjing 210023, China; Department of Human Anatomy and Histoembryology, Nanjing University of Chinese Medicine, Nanjing 210023, China

**Keywords:** lateral parabrachial nucleus, central nucleus of the amygdala, cholinergic projections, methamphetamine priming-induced reinstatement of conditioned place preference

## Abstract

Drug relapse is a big clinical challenge in the treatment of addiction, but its neural circuit mechanism is far from being fully understood. Here, we identified a novel cholinergic pathway from choline acetyltransferase-positive neurons in the external lateral parabrachial nucleus (eLPB^ChAT^) to the GABAergic neurons in the central nucleus of the amygdala (CeA^GABA^) and explored its role in methamphetamine priming-induced reinstatement of conditioned place preference. The anatomical structure and functional innervation of the eLPB^ChAT^–CeA^GABA^ pathway were investigated by various methods such as fluorescent micro-optical sectioning tomography, virus-based neural tracing, fibre photometry, patch-clamp and designer receptor exclusively activated by a designer drug. The role of the eLPB^ChAT^–CeA^GABA^ pathway in methamphetamine relapse was assessed using methamphetamine priming-induced reinstatement of conditioned place preference behaviours in male mice. We found that the eLPB^ChAT^ neurons mainly projected to the central nucleus of the amygdala. A chemogenetic activation of the eLPB^ChAT^ neurons *in vitro* or *in vivo* triggered the excitabilities of the CeA^GABA^ neurons, which is at least in part mediated via the cholinergic receptor system. Most importantly, the chemogenetic activation of either the eLPB^ChAT^ neurons or the eLPB^ChAT^ neurons that project onto the central nucleus of the amygdala decreased the methamphetamine priming-induced reinstatement of conditioned place preference in mice. Our findings revealed a previously undiscovered cholinergic pathway of the eLPB^ChAT^–CeA^GABA^ and showed that the activation of this pathway decreased the methamphetamine priming-induced reinstatement of conditioned place preference.

## Teaser

He *et al*. identified a novel cholinergic projection from choline acetyltransferase-positive neurons in the external lateral parabrachial nucleus (eLPB^ChAT^) to the GABAergic neurons in the central nucleus of the amygdala (CeA^GABA^), which is involved in methamphetamine relapse. The chemogenetic activation of either the LPB^ChAT^ neurons or the eLPB^ChAT^–CeA^GABA^ pathway attenuated the methamphetamine-primed reinstatement of conditioned place preference.

## Graphical Abstract

**Graphical abstract:**
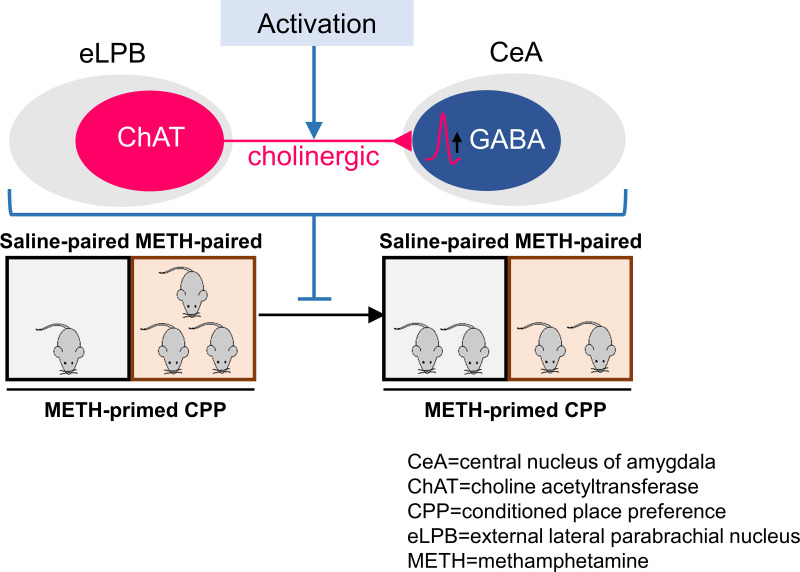

## Introduction

Methamphetamine (METH) is one of the most commonly abused drugs in the world. Drug relapse is a big clinical challenge in the treatment of addiction, but its neural circuit mechanism is far from fully understood. The lateral parabrachial nucleus (LPB) is located at the boundary of the pontine and midbrain, lateral to the superior cerebellar peduncle (scp). According to the anatomical position, LPB can be further subdivided into the dorsal lateral parabrachial nucleus (dLPB) and external lateral parabrachial nucleus (eLPB). The LPB neurons that express calcitonin gene-related peptide-expressing neurons (LPB^CGRP^) or glutamate neurons (LPB^GLU^) have been well-studied in the processes of reward,^1^ food intake,^2,3^ emotion and mental disorders including addiction.^4–7^ In 1993, Bechara *et al*.^6^ showed that LPB lesions blocked conditioned taste aversion (CTA) produced by low intraperitoneal doses of morphine in rats. Subsequently, both morphine and cocaine administration in rats,^5^ as well as naloxone-precipitated withdrawal in morphine rats,^7^ induced significantly increased levels of LPB c-Fos. Recently, Lin *et al*.^4^ reported that morphine administration activated a glutamatergic pathway from the LPB to the dorsal raphe (DRN), while blocking the LPB neurotransmission ultimately reduced the morphine-induced conditioned place preference (CPP) expression in mice, indicating a critical role of the LPB neurons in addictive behaviours. However, few studies in the literature have reported the role of LPB in drug relapse. Noteworthily, a recent study found that choline acetyltransferase (ChAT)-positive neurons (LPB^ChAT^) exist in the LPB,^8^ yet their projections and functions have not been explored.

The central nucleus of the amygdala (CeA) is one of the main nuclei that receives projections from the LPB.^9,10^ The CeA neurons express nicotinic acetylcholine receptors (nAChRs)^11,12^ and receive cholinergic projections.^13,14^ Functionally, the CeA is considered to be a key region associated with drug relapse, including incubation (drug-seeking progressively increases after prolonged withdrawal from extended access to METH) of METH-seeking behaviours,^15^ cue-induced reinstatement of METH-seeking behaviours^16^ and stress-induced reinstatement of cocaine-seeking behaviours.^17^ Optogenetic activation of LPB projections in the CeA decreases food intake^3^ as well as CTA.^18^ A recent review hypothesized that LPB-extended amygdala circuits process interoceptive and exteroceptive stimuli, which may in part contribute to the dysregulated affective state induced by abstinence from chronic drug use.^19^

In the present study, we dissected a novel cholinergic pathway from eLPB^ChAT^ neurons and explored its role in METH priming-induced reinstatement of CPP in male mice. Reinstatement is a classical extinction-based drug relapse model^20^ that refers to the resumption of drug-seeking behaviours after extinction following exposure to drugs, drug-associated cues or contexts, or stressors.^21^

## Materials and methods

Detailed experimental methods are provided in Supplementary Materials.

### Mice

C57BL/6 wild type (WT) and ChAT-Cre male mice weighing 25–35 g were used.

### Immunofluorescence

The following primary antibodies were used: goat polyclonal anti-ChAT (1:200, RRID: AB_2079751, Millipore, USA), rabbit polyclonal anti-NeuN (1:200, RRID: AB_2651140, Cell Signaling Technology, USA) and rabbit polyclonal anti-c-Fos (1:1500, RRID: AB_2247211, Cell Signaling Technology, USA).

### Tracing virus injection

All virus samples in the present study were packaged by BrainVTA (Wuhan, China). The following virus samples were used: *rAAV2/9-ChAT-EGFP* (PT-1722, 2.15E+12 vg/ml), *rAAV2/9-EF1α-DIO-EGFP* (PT-0795, 2.04E+12 vg/ml), *CTB-555* (CTB-02, 1 μg/μl), *rAAV2/9-VGAT1-CRE* (PT-0346, 3.63E+12 vg/ml, 25 nl), *rAAV2/9-DIO-TVA* (PT-0021, 5.56E+12 vg/ml, 25 nl), *rAAV2/9-DIO-RVG* (PT-0023, 5.29E+12 vg/ml, 50 nl), *RV-EnvA-ΔG-DsRed* (R01002, 2.00E+08 IFU/ml), *rAAV2/9-ChAT-hM3Dq-mCherry* (PT-2213, 5.54E+12 vg/ml) and *rAAV2/9-VGAT1-EGFP* (PT-3176, 5.31E+12 vg/ml).

Unless otherwise noted, a volume of 100 nl of virus sample was injected per side. The following stereotaxic coordinates for the eLPB are used: AP, −5.20 mm; ML, ±1.55 mm and DV, −3.60 mm. The following stereotaxic coordinates of the CeA are used: AP, −0.90 mm; ML, ±2.70 mm; DV, −4.55 mm.

### Fluorescent micro-optical sectioning tomography

In fluorescent micro-optical sectioning tomography (fMOST), the *rAAV2/9-ChAT-EGFP* (PT-1722, 2.15E+12 vg/ml) virus was unilaterally injected into the eLPB of the WT mice. The intact brains were mapped using BioMapping 5000N (Oebio, Wuhan, China).

### Designer receptor exclusively activated by designer drug

Clozapine N-oxide (CNO, 2 mg/kg,^22^ HY-17366, MedChemExpress) was used to specifically modulate the eLPB^ChAT^ neurons via interaction with the *hM3Dq* virus for 30 min before performing behavioural tests.^23^

### Patch-clamp

In the patch-clamp experiment, *rAAV2/9-ChAT-hM3Dq-mCherry* (PT-2213, 5.54E+12 vg/ml) was bilaterally injected into the eLPBs, followed by *rAAV2/9-VGAT1-EGFP* (PT-3176, 5.31E+12 vg/ml) being bilaterally delivered into the CeAs of WT mice.

Preparation of slices was done as previously described.^24^ The spontaneous action potentials (sAPs) were recorded under the current-clamp mode, while the spontaneous excitatory postsynaptic currents (sEPSCs) were recorded under the voltage-clamp (voltage holding at −70 mV) mode. 10 μM of CNO^23^ was used to activate the terminals of the eLPB^ChAT^ neurons within the CeA. 50 µM of picrotoxin^25^ was used to block the GABA_A_ receptors. 5 μM of mecamylamine (MEC)^26^ was used to non-specifically inhibit nAChRs on the CeA^GABA^ neurons.

### Fibre photometry

The *rAAV2/9-VGAT1-GCaMp6m* (PT-3317) virus was bilaterally injected into the CeAs, and the *rAAV2/9-ChAT-hM3Dq* (Gq, PT-2874, 5.54E+12 vg/ml) or *rAAV2/9-ChAT* (Go, PT-0607, 5.50E+12 vg/ml) virus was bilaterally injected into the eLPBs of WT mice. The calcium signals were obtained by stimulating these cells with a 405 nm LED (15–20 μW at the fibre tip). F0 is the baseline fluorescence signal that was recorded for 1 min prior to CNO treatment. F is the real-time fluorescence signal that was recorded at 0–50 min. The values of Δ*F*/*F* are calculated by (F–F0)/F0. The area under curve (AUC) is the integral under recording duration related to the corresponding baseline at every trial.

### Conditioned place preference

In the CPP experiment, two cohorts of WT mice were exposed to viral injections. In Cohort-1 mice, *rAAV2/9-ChAT-hM3Dq-mCherry* (Gq, PT-2213, 5.54E+12 vg/ml) or *rAAV2/9-ChAT* (Go, PT-0607, 5.50E+12 vg/ml) was bilaterally injected into the eLPBs, forming eLPB-Gq mice and eLPB-Go mice. In Cohort-2 mice, *rAAV2/9-ChAT-DIO-hM3Dq-mCherry* (Gq, PT-2825, 5.08E+12 vg/ml) or *rAAV2/9-ChAT* (Go, PT-0607, 5.50E+12 vg/ml) was bilaterally delivered into the eLPBs, followed by *rAAV2/retro-Cre-EGFP* (PT-1168, 5.25E+12 vg/ml, 150 nl) being bilaterally injected into the CeAs, forming CeA-Gq mice and CeA-Go mice. Mice received CNO (2 mg/kg, i.p.) 30 min before each behavioural test.

METH CPP procedures were performed using the TopScan3D CPP apparatus (CleverSys, VA, USA). A standard CPP protocol^21,27^ was applied, including a pre-test, conditioning, a post-conditioning test, extinction training and a METH challenge-primed reinstatement test. Baseline preference (pre-test) was assessed by placing the mice in a random chamber of the CPP apparatus and allowing them to explore all two chambers freely. Conditioning was confined to a preferred chamber paired with a saline (0.2 ml, i.p.) injection in the morning and to a non-preferred chamber paired with a METH (3 mg/kg, i.p.) injection in the afternoon for 7 consecutive days. During the test and extinction, mice were allowed to freely access the two chambers without any injections. For the METH-primed reinstatement test, mice were injected with METH (0.5 mg/kg, i.p.) and then allowed to freely explore both chambers for 15 min.

The CPP score was calculated by subtracting the duration spent in the saline-paired chamber from the METH-paired chamber, and the ΔCPP score was the reinstatement CPP score minus the extinction CPP score.

### Statistical analysis

Statistical analysis was carried out using GraphPad Prism 8.0 software. The paired *t*-tests, unpaired *t*-tests and repeated measures of two-way ANOVA with *Sidak post hoc* tests were used to analyse data. Statistical significance was set as *P* < 0.05.

### Data availability

The data that support the findings of this study are available from the corresponding author upon reasonable request.

## Results

### Anatomical dissection of the eLPB^ChAT^–CeA^GABA^ pathway

**Figure 1:**
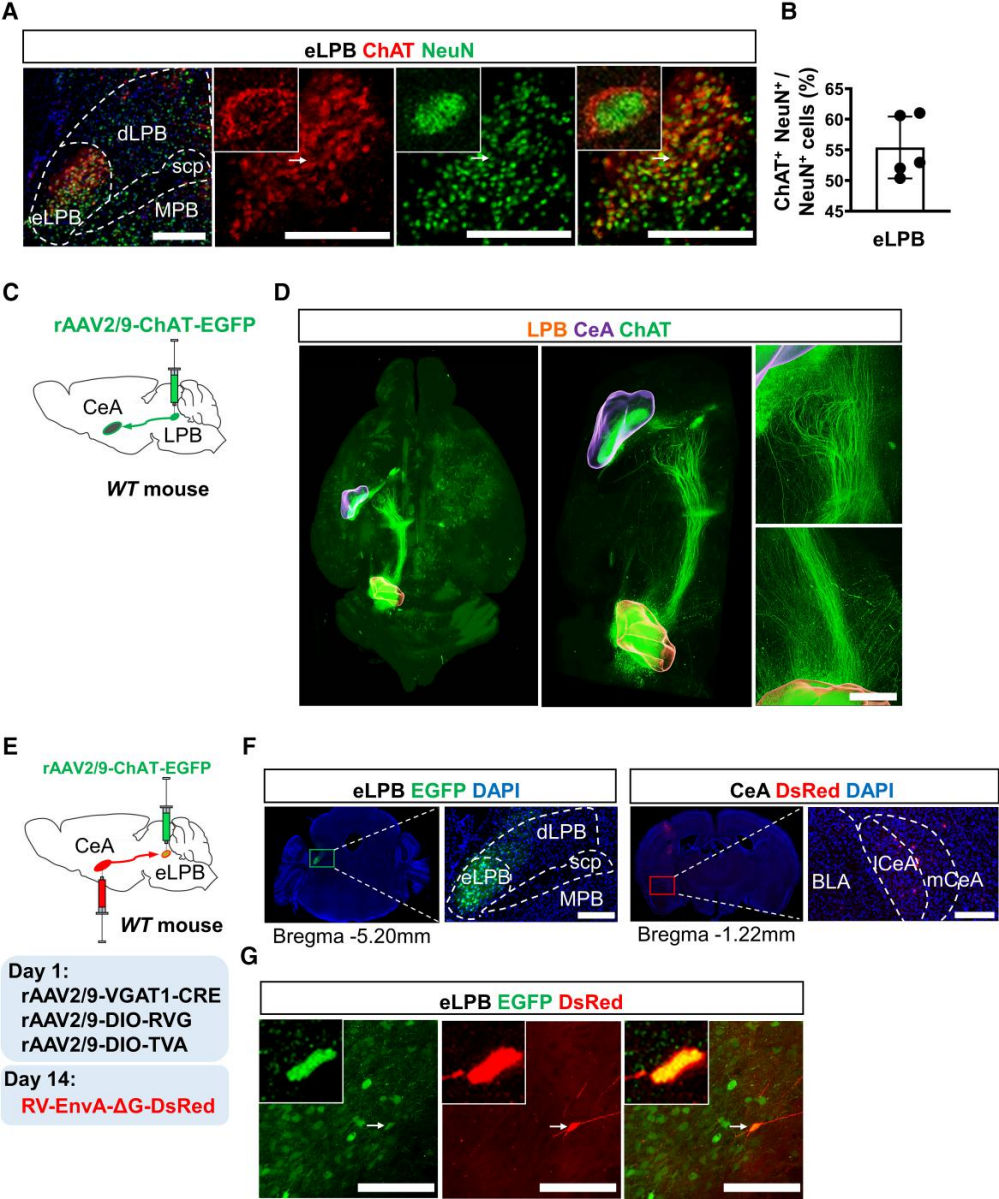
**Anatomical structure of the eLPB^ChAT^–CeA^GABA^ pathway.** (**A**) Immunohistochemistry for ChAT/NeuN in the eLPB of WT mice. Scale bar, 400 μm. (**B**) The percentage of ChAT-positive cells relative to NeuN-labelled cells in the eLPB, *n* = 5 mice. (**C**) Schematic diagram of the *rAAV2/9-ChAT-EGFP* injection in WT mice. (**D**) The overview of the cholinergic projections from LPB^ChAT^ neurons in the brain by fMOST. Scale bar, 50 pixels. (**E**) Schematic diagram of viral injection sites in the eLPB and CeA of WT mice. (**F**) Representative images of *rAAV2/9-ChAT-EGFP* injection into the eLPB and *RV-EnvA-ΔG-DsRed* injection into the CeA. Scale bar, 400 μm. (**G**) Representative images of EGFP-labelled and DsRed-labelled viral signals that are co-expressed within the eLPB. Scale bar, 100 μm.

First, we dissected the anatomical structure of the potential cholinergic pathway from the eLPB^ChAT^ to the CeA^GABA^. The neuronal nuclear antigen (NeuN) and ChAT were used as specific markers for neurons and cholinergic neurons, respectively. As shown in Fig. 1A, ChAT-positive neurons were mainly located in the eLPB. Immunohistochemical analysis revealed that >50% of the eLPB neurons were ChAT-positive neurons (eLPB^ChAT^, Fig. 1B). To overview the whole-brain atlas of direct eLPB^ChAT^ projections, whole-brain precise imaging was performed by fMOST by injecting *rAAV2/9-ChAT-EGFP* into the eLPB of WT mice to label the eLPB^ChAT^ and the axonal projections (Fig. 1C). As shown in Fig. 1D, Supplementary Fig. 1A and Video 1, the eLPB^ChAT^ represented particularly strong inputs to the ipsilateral CeA, delineating the previously undiscovered eLPB^ChAT^–CeA pathway.

To exclude the possibility that the CeA inadvertently labelled eLPB^ChAT^ fibres passing through rather than synapsing on the CeA, and further to determine the monosynaptic inputs from the eLPB^ChAT^ to the CeA^GABA^, anterograde trans-synaptic rabies tracing was used in combination with Cre-dependent version (Fig. 1E). The eLPB^ChAT^ neurons were infected by a ChAT promoter-driven virus expressing EGFP, while CeA^GABA^ neurons as the starter cells (VGAT1-Cre and two Cre-dependent AAV helper virus recombinants in the CeA) were infected by rabies virus expressing DsRed (Fig. 1F). As shown in Fig. 1G, rabies virus-labelled neurons (DsRed-positive) in the eLPB were co-expressed with *ChAT*-transfected eLPB^ChAT^ neurons (EGFP-positive), indicating a direct pathway from the eLPB^ChAT^ to the CeA^GABA^.

To accurately describe and quantify the eLPB^ChAT^–CeA pathway in ChAT-Cre mice, a Cre-dependent anterograde tracing virus labelled with EGFP was injected into the eLPB (Supplementary Figure 1B). Immunohistochemical analysis revealed that most of the EGFP-labelled eLPB neurons were also immune-positive for ChAT (Supplementary Fig. 1C), and the eLPB^ChAT^ sent branching axons to the lateral region of the CeA (lCeA, Supplementary Fig. 1D). In WT mice, retrograde tracing *CTB-555* was injected into the CeA (Supplementary Fig. 1E, 1F). Immunohistochemical analysis showed that, in the eLPB, around 27% of the ChAT-positive neurons were co-labelled with *CTB-55*5 retrograded from the CeA, and 68% of *CTB-555* were co-expressed with the ChAT-positive neurons (Supplementary Fig. 1G, H). Together, we found a novel direct cholinergic pathway from the eLPB^ChAT^ to the CeA^GABA^, forming an eLPB^ChAT^–CeA^GABA^ pathway.

### Functional investigation of the eLPB^ChAT^–CeA^GABA^ pathway

**Figure 2:**
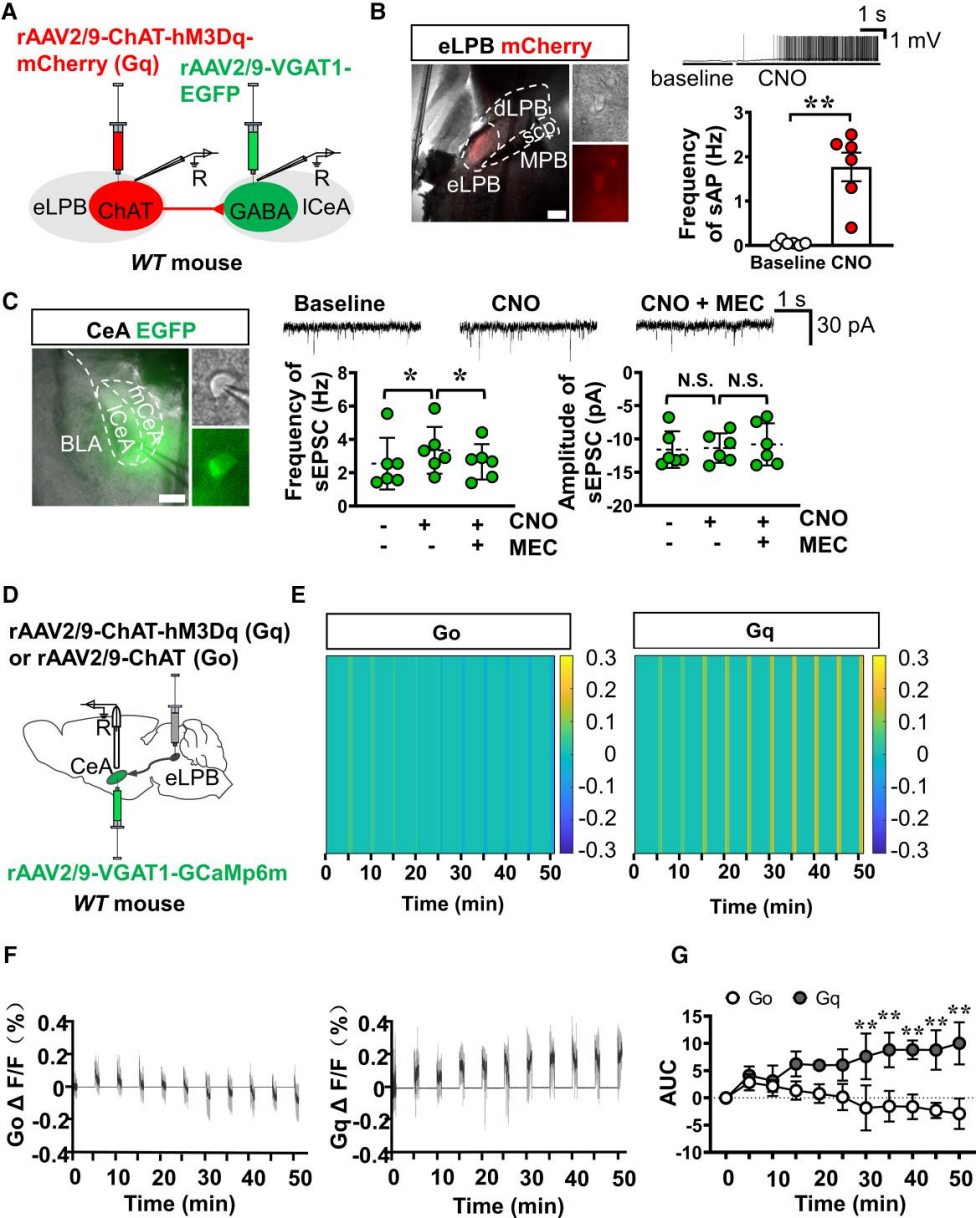
**Functional role of the eLPB^ChAT^–CeA^GABA^ pathway.** (**A**) Schematic diagram of the viral transfection in WT mice and the patch-clamp recording in brain slices. (**B**) Representative images of current-clamp recording on *rAAV2/9-ChAT-hM3Dq-mCherry* (*Gq*) transfected neurons in the eLPB (left), sample traces and summarized data of sAP in eLPB^ChAT^ neurons (right) after CNO treatment. *n* = 6 cells from six mice. Scale bar, 400 μm. (**C**) Representative images of patch-clamp recording on the *rAAV2/9-VGAT1-EGFP* transfected neurons in the CeA (left), sample traces and summarized data of frequency (middle, *n* = 6 cells from six mice, one-way ANOVA with *Tukey post-test*) and amplitude (right, *n* = 6 cells from six mice, one-way RM ANOVA with Tukey post-test) of sEPSC in response to CNO and MEC. Scale bar, 400 μm. (**D**) Schematic diagram of viral transfection and optical fibre implantation in the eLPB and CeA. (**E**) Heatmap of *GCaMp6m* fluorescence at 0–50 min after CNO administration. (**F**) Quantification of the peak Δ*F*/*F*. (**G**) Go and Gq-evoked AUC. *n* = 6 mice, two-way ANOVA with *Sidak post-test*.

To characterize the functional innervation of the eLPB^ChAT^–CeA^GABA^ pathway, we combined neuronal activator designer receptor exclusively activated by designer drug (DREADD) hM3D and patch-clamp recording in acutely prepared slices. *ChAT-hM3Dq* (*Gq*) virus labelled with mCherry was injected into the bilateral eLPBs to infect the eLPB^ChAT^ neurons, and *VGAT1* promoter-driven virus labelled with EGFP was injected into the CeA to transfect the CeA^GABA^ neurons (Fig. 2A). CNO (CNO) was used to chemogenetically activate neurons by interaction with the hM3Dq (*Gq)* virus. As shown in Fig. 2B, the frequency of sAP in the eLPB^ChAT^ neurons was significantly increased by bath application of CNO when compared with its baseline, indicating the successful *Gq* virus models (*t* = 5.088, df = 5, **P = 0.0038 versus baseline). Next, Fig. 2C showed that a stimulation of hM3Dq (*Gq*)-expressed eLPB^ChAT^ terminals on the CeA slices by CNO increased the frequency of sEPSCs in the CeA^GABA^ neurons. In addition, the enhanced sEPSC frequency disappeared when CNO was washed out from bath artificial cerebrospinal fluid (ACSF), and the CNO-enhanced sEPSC frequency was blocked by a non-specific nAChRs antagonist (MEC) (*F*_(1.597, 7.986)_ = 6.229, *P* = 0.0275, *P = 0.0464 CNO versus baseline, *P = 0.0325 MEC versus CNO). However, both CNO and MEC treatment had no effect on the amplitude of sEPSC (*F*_(1.408, 7.039)_ = 0.2703, *P* = 0.6966, ^N.S.^*P* = 0.9756 CNO versus baseline, ^N.S.^*P* = 0.7498 MEC versus CNO). These results indicate that the activation of eLPB^ChAT^ neurons is necessary and sufficient to excite CeA^GABA^ neurons, and which was at least in part via nAChRs.

To further confirm the innervation of the eLPB^ChAT^ on the CeA^GABA^ *in vivo*, real-time calcium signals in free-moving mice were recorded in the CeA^GABA^ neurons by injecting the *VGAT1-GCaMp6m* virus into the CeA, and the ChAT promoter-driven hM3Dq (*Gq*) or ChAT alone (*Go*) virus was injected into the bilateral eLPB in WT mice (Fig. 2D). The GCaMp6m-positive virus was expressed restrictedly in the CeA and was highly overlapping with GAD67 (Supplementary Fig. 2A,B) (*t* = 0.8530, df = 4, ^N.S.^*P* = 0.4417 versus Go). As shown in Fig. 2E, F and G, after the chemogenetic activation of the eLPB^ChAT^ neurons by a systemic administration of CNO, sustained increases in calcium signals at 30, 35, 40, 45 and 50 min were observed in the GCaMp6m-transfected CeA^GABA^ neurons (*F*_(4, 40)_ = 9.687, *P* < 0.0001; **P_(30 min)_ = 0.0004, **P_(35 min)_ < 0.0001, **P_(40 min)_ < 0.0001, **P_(45 min)_ < 0.0001, ***P_(50 min)_ = <0.0001 versus Go.). In addition, immunohistochemical analysis revealed that the systemic administration of CNO significantly evoked c-Fos expression in the CeA^GABA^ neurons expressing GCaMp6m (Supplementary Fig. 2C,D) (*t* = 3.441, *df* = 4, *P=0.0263 versus Go). These results confirmed the positive innervation of the eLPB^ChAT^–CeA^GABA^ pathway under physiological conditions.

Taken together, we found that the specific activation of the eLPB^ChAT^ neurons *in vitro* or *in vivo* excited the CeA^GABA^ neurons. In addition, the nAChRs antagonist effectively inhibited the CNO-enhanced sEPSC in the CeA^GABA^ neurons with *Gq*-transfected terminals from the eLPB^ChAT^ neurons.

### Effect of the chemogenetic activating eLPB^ChAT^–CeA^GABA^ pathway on METH priming-induced reinstatement of CPP

**Figure 3:**
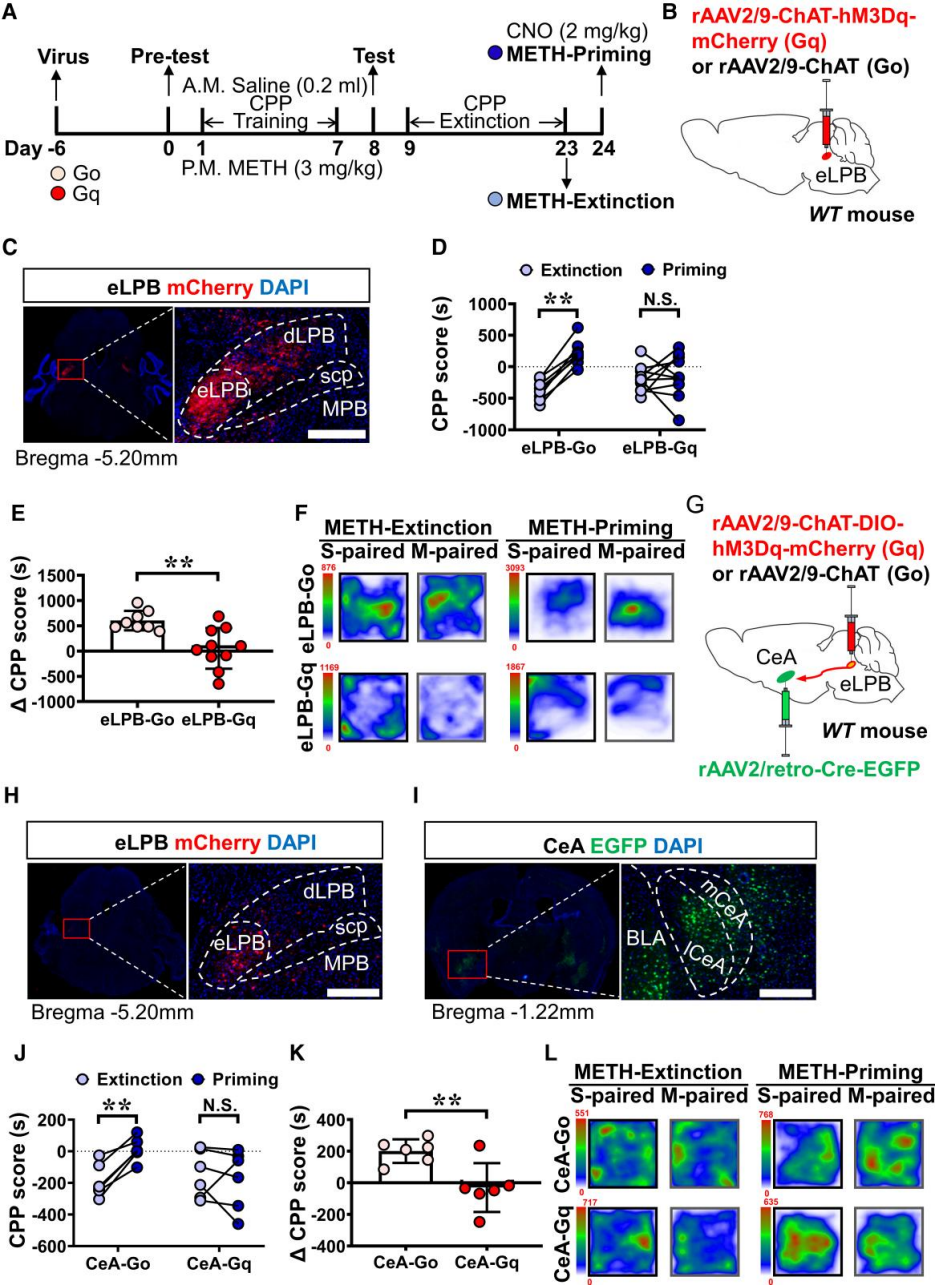
**The role of the eLPB^ChAT^–CeA^GABA^ pathway in METH priming-induced reinstatement of CPP.** (**A**) Experimental design and timeline of METH priming-induced reinstatement of CPP. (**B**) Schematic diagram of the viral transfection in WT mice. (**C**) Representative images of *rAAV2/9-ChAT-hM3Dq-mCherry* (*Gq*) injection in the eLPB. Scale bar, 400 μm. (**D and E**), The METH priming-induced reinstatement of CPP after activating eLPB^ChAT^ neurons by CNO. eLPB-Go: *n* = 8 mice; eLPB-Gq: *n* = 10 mice; ΔCPP scores, *n* = 18. (**F**) The heatmap of mice travelling traces in eLPB-Go and eLPB-Gq mice. (**G**) Schematic diagram of the viral transfection in the eLPB and CeA of WT mice. (**H**) Representative images of *rAAV2/9-ChAT-hM3Dq-mCherry* (*Gq*) injection in the eLPB. Scale bar, 400 μm. (**I**) Representative images of *rAAV2/retro-Cre-EGFP* within the CeA. Scale bar, 400 μm. **(J and K)** METH priming-induced reinstatement of CPP after activating terminals from eLPB^ChAT^ neurons within the CeA. CeA-Go: *n* = 6 mice; CeA-Gq: *n* = 6 mice); ΔCPP scores, *n* = 12 mice. (**L**) The heatmap of mice travelling traces in CeA-Go and CeA-Gq mice.

To investigate the role of eLPB^ChAT^ neurons in METH relapse, the METH priming-induced reinstatement of the CPP procedure was set up in mice (Fig. 3A). Before METH CPP training, the ChAT promoter-driven hM3Dq (*Gq*) or ChAT alone (*Go*) virus was injected into the bilateral eLPBs in WT mice (Fig. 3B, C, eLPB-Gq and eLPB-Go mice, respectively). The mCherry-positive virus was expressed restrictedly in the eLPB and was highly overlapping with ChAT (Supplementary Fig. 3A, B). As shown in Supplementary Fig. 3C–F, no significant differences can be found on the METH-induced CPP (*F*_(1, 16)_ = 6.234, *P* = 0.0238. eLPB-Go, baseline versus test: **P = 0.0091; eLPB-Gq, baseline versus test: **P<0.0001) and CPP extinction training (*F*_(14, 224)_ = 1.473, *P* = 0.1224) between two groups. During the priming test on D24, a single challenge of low-dose METH successfully reinstated the METH-induced CPP in eLPB-Go mice but failed to reinstate it in eLPB-Gq mice after the systemic administration of CNO (eLPB-Go: *t* = 10.60, *df* = 7, **P <0.0001 versus extinction; eLPB-Gq: *t* = 0.4251, *df* = 9, ^N.S.^*P* = 0.6808 versus extinction; ΔCPP scores, *t* = 3.546, *df* = 16, **P = 0.0027 versus Go, Fig. 3D–F). In contrast, the total distance travelled by the mice between the eLPB-Gq and the eLPB-Go models was not significantly different (*t* = 0.3193, *df* = 16, ^N.S.^*P* = 0.7536 versus eLPB-Go, Supplementary Fig. 3E). These results indicated that the activation of the eLPB^ChAT^ decreased the METH priming-induced reinstatement of CPP without changing the locomotive abilities in the mice.

To evaluate the role of the eLPB^ChAT^–CeA^GABA^ pathway in the METH priming-induced reinstatement of CPP, we expressed Cre recombinase in the CeA neurons by injecting *Raav2/Retro-Cre-EGFP* into the bilateral CeA and infected CeA-projecting Elpb^ChAT^ neurons bilaterally with *AAV2/9-ChAT-DIO-Hm3Dq-mCherry (Gq)* or *ChAT alone* (*Go*) in WT mice (Fig. 3G–I, CeA-Gq and CeA-Go mice, respectively). As shown in Fig. 3J–L, the chemogenetic activation of the eLPB^ChAT^ neurons projecting to the CeA neurons obviously decreased the METH priming-induced reinstatement of CPP in CeA-Gq mice, when compared with that in CeA-Go mice (CeA-Go: *t* = 6.579, *df* = 5, **P = 0.0012 versus extinction; CeA-Gq: *t* = 0.6573, *df* = 5, ^N.S.^*P* = 0.6573 versus extinction; ΔCPP scores, *t* = 3.286, *df* = 10, **P = 0.0082 versus Go.). As shown in Supplementary Fig. 3F and G, no significant differences were observed during the CPP test (*F*_(1, 10)_ = 0.06524, *P* = 0.8036. CeA-Go, Test: *P = 0.0120 versus Baseline; CeA-Gq, **P = 0.0066, versus Baseline) and extinction training (*F*_(14, 140)_ = 0.7511, *P* = 0.7196) between the two groups. There was no significant difference in the total distance travelled by the mice between the CeA-Gq and CeA-Go models (*t* = 0.6060, *df* = 10, ^N.S.^*P* = 0.5580 versus CeA-Go, Supplementary Fig. 3H). These results indicated that the activation of the eLPB^ChAT^–CeA pathway effectively decreased the METH priming-induced reinstatement of CPP without changing the locomotive abilities in the mice.

## Discussion

The cholinergic neurons play critical roles in processing reward- and addiction-related information,^28–32^ and cholinergic dysfunction leads to neurological and psychiatric disorders.^33^ Cholinergic neurons in the mammalian brain are thought to be mainly distributed in five regions, namely the pedunculopontine, dorsal lateral tegmental nucleus, thalamic nucleus, striatum and basal forebrain nucleus.^34^ ChAT-positive neurons can either serve as interneurons locally or send out long-distance projections to control other brain regions.^28,29^ For example, regulating cholinergic interneurons in the nucleus accumbens (NAc) suppressed cocaine CPP, cocaine self-administration, as well as cue-induced reinstatement of heroin-seeking.^35,36^ However, a question arises as to whether the cholinergic system, especially the cholinergic projecting neuron, contributes to METH addiction. In 1999, Kish *et al*.^37^ found that exposure to high doses of METH caused brain ChAT depletion in autopsied brain of chronic METH users. Subsequently, they further found that vesicular acetylcholine transporter (VAChT, a ‘stable’ marker of human cholinergic neurons) levels were selectively elevated by 48% in the METH group.^38^ Until recently, with the ChAT-Cre transgenic mice, Nasirova *et al*.^8^ reported that ChAT-positive neurons existed in the LPB of mouse embryo. Consistent with this finding, we found that there existed abundant LPB^ChAT^ neurons in adult mice, which are concentrated in the eLPB. Most importantly, the specific activation of LPB^ChAT^ decreased METH-primed CPP behaviours, indicating the critical role of the eLPB^ChAT^ in the METH priming-induced reinstatement of CPP.

The CeA is one of the major LPB afferent sources and efferent targets.^10^ Some studies showed that the CeA mainly received inputs from CGRP-positive neurons ^31,39,40^ or pituitary adenylate cyclase-activating polypeptide (PACAP) neurons^41,42^ in the LPB, most of which were glutamatergic neurons.^43,44^ Do eLPB^ChAT^ neurons send cholinergic projections directly to the CeA? Here, we found that there exists a direct cholinergic eLPB^ChAT^–CeA^GABA^ pathway, which extends the knowledge of classic LPB–CeA circuits. It is possible that some neurons in the LPB co-express the ACh with glutamate, which is akin to many LPB neurons expressing CGRP with glutamate.^8^ ACh plays a role in the establishment or refinement of glutamatergic synaptic connections,^45,46^ which would allow ACh to act homosynaptically in synapse maturation and plasticity. Here, we illustrated that DREADD-mediated activation of eLPB^ChAT^ neuron projection into the CeA^GABA^ neurons increased the frequency of sEPSCs *in vitro* and triggered the calcium signal *in vivo* in the CeA^GABA^ neurons, indicating an exciting innervation effect of the eLPB^ChAT^ on the CeA^GABA^ neurons. Further, the nAChRs antagonist reversed the increases of sEPSCs, indicating that the positive innervation of the eLPB^ChAT^ neurons on CeA^GABA^ was mediated at least in part via cholinergic projections.

The CeA contains 95% GABAergic medium-sized neurons.^47^ Studies have shown that the inactivation of the CeA by GABA agonism blocked stress-induced reinstatement of cocaine-seeking.^17,48,49^ Moreover, reversible inactivation (lidocaine or GABAA and GABAB receptor agonists) of the CeA decreased cue-induced reinstatement of METH-seeking after extinction.^16,50^ Consistent with previous studies, we found that the activation of the eLPB^ChAT^ neurons projecting onto the CeA decreased the METH priming-induced reinstatement of CPP in male mice, supporting the concept that the CeA is critical for drug relapse. The lCeA receives abundant LPB^ChAT^ projections, which exits two types of non-over-lapping but mutually suppresses GABA neurons, expressed with protein kinase C-δ (PKCδ) or somatostatin (SOM), respectively.^51,52^ The lCeA^PKCδ^ and lCeA^SOM^ neurons have opposite effects on the output neurons in the medial region of the CeA (mCeA): The lCeA^PKCδ^ neurons inhibit these output neurons that promote aversive behaviour, while lCeA^SOM^ neurons promote motivated behaviour by disinhibiting these output neurons.^53,54^ Venniro *et al*.^55^ demonstrated that METH-forced abstinence increased Fos expression in both lCeA^PKCδ^ and lCeA^SOM^. It is not known whether and how the two types of lCeA neurons contribute to METH priming-induced reinstatement of CPP. They further identified that social choice-induced voluntary abstinence decreased METH craving, which was mediated by the activation of lCeA^PKCδ^. In contrast, incubation after forced abstinence promoted METH craving, which was mediated by the activation of lCeA^SOM^.^56^ In the present study, our data showed that activating LPB^ChAT^ neurons in whole or those projecting to the CeA^GABA^ decreased METH-primed CPP in mice, suggesting the important role of the eLPB^ChAT^–CeA^GABA^ pathway in METH priming-induced reinstatement of CPP. A further study should dissect the roles of the eLPB^ChAT^–CeA^PKCδ^ and/or the eLPB^ChAT^–CeA^SOM^ pathway in the process of METH priming-induced reinstatement of CPP in mice.

There are some limitations in the present study. First, it is important for reinstatement studies to consider not only drug priming, but also the extinction response in the absence of a reinstating stimulus. Also, it needs to be ascertained whether the manipulation of the eLPB^ChAT^–CeA^GABA^ pathway could induce reinstatement behaviours during the process of extinction training. Second, the molecules in the eLPB^ChAT^–CeA^GABA^ pathway that contribute to METH-primed reinstatement of CPP are required to be explored in a future study.

In summary, we identified a novel cholinergic pathway from the eLPB^ChAT^ neurons to the CeA^GABA^ neurons, forming the eLPB^ChAT^–CeA^GABA^ pathway. Under physiological conditions, the activation of the eLPB^ChAT^ neurons or their terminals on the CeA^GABA^ neurons triggered the excitability of these CeA^GABA^ neurons. Under the METH priming-induced reinstatement of CPP, activating either the eLPB^ChAT^ neurons in whole or in the eLPB^ChAT^–CeA^GABA^ pathway decreased the METH-primed CPP in mice, indicating that the eLPB^ChAT^–CeA^GABA^ pathway is involved in coding the process of METH priming-induced reinstatement of CPP.

## Supplementary Material

fcac219_Supplementary_Data

## Abbreviations

AUC =: area under curve
CeA =: central nucleus of the amygdala
ChAT =: choline acetyltransferase
CNO =: Clozapine N-oxide
CPP =: conditioned place preference
CTA =: conditioned taste aversion
dLPB =: dorsal lateral parabrachial nucleus
DREADD =: designer receptor exclusively activated by designer drug
DRN =: dorsal raphe
eLPB =: external lateral parabrachial nucleus
fMOST =: fluorescent micro-optical sectioning tomography
GLU =: glutamate
lCeA =: lateral region of CeA
LPB =: lateral parabrachial nucleus
mCeA =: medial region of CeA
MEC =: mecamylamine
METH =: methamphetamine
NAc =: nucleus accumbens
nAChRs =: nicotinic acetylcholine receptors
NeuN =: neuronal nuclear antigen
PACAP =: pituitary adenylate cyclase-activating polypeptide
PKCδ =: protein kinase C-δ
sAP =: spontaneous action potentials
scp =: superior cerebellar peduncle
sEPSC =: spontaneous excitatory postsynaptic currents
SOM =: somatostatin
VAChT =: vesicular acetylcholine transporter
WT =: wild type
