# Supplementary material for: A novel cholinergic projection from the lateral parabrachial nucleus and its role in methamphetamine-primed conditioned place preference
Source: Brain Commun. 2022 Aug 30;4(5):fcac219. doi: 10.1093/braincomms/fcac219 (PMC9536296; doi:10.1093/braincomms/fcac219)
Supplement: fcac219_Supplementary_Data [file fcac219_Supplementary_Data.zip › Suppemental_Methods.docx]

**Supplementary Methods**

**Animals**

C57BL/6 wild type (WT) and ChAT-Cre male mice weighing 25-35 g were used. Animals were housed at constant humidity (40-60%) and temperature (24 ± 2 °C) with a 12-hour light/dark cycle, and allowed free access to food and water. All procedures were carried out in accordance with the National Institutes of Health Guide for the Care and Use of Laboratory Animals and approved by the Institutional Animal Care and Use Committee (IACUC) at Nanjing University of Chinese Medicine.

**Immunofluorescence**

The mice were deeply anesthetized with 10% chloral hydrate (0.2 ml, i.p.) and sequentially perfused with 0.9% saline and 4% PFA. The brains were removed and post-fixed in 4% PFA at 4 °C overnight. After cryoprotection of the brains with 30% (w/v) sucrose, coronal sections (30 μm) were cut on a cryostat (Leica, Germany) and used for immunofluorescence. The sections were incubated with the following primary antibodies: goat polyclonal anti-ChAT (1:200, RRID: AB_2079751, Millipore, USA), rabbit polyclonal anti-NeuN (1:200, RRID: AB_2651140, Cell Signaling Technology, USA) and rabbit polyclonal anti-c-Fos (1:1500, RRID: AB_2247211, Cell Signaling Technology, USA) at 4 °C for 24 h, followed by the corresponding fluorophore-conjugated secondary antibodies for 2 h at room temperature. The following secondary antibodies were used here: Alexa Fluor 555-labeled donkey anti-goat secondary antibody (1:500, RRID: AB_2762839, Invitrogen, USA), Alexa Fluor 488-labeled donkey anti-goat secondary antibody (1:500, RRID: AB_2762838, Invitrogen, USA), Alexa Fluor 488-labeled donkey anti-rabbit secondary antibody (1:500, RRID: AB_2762833, Invitrogen, USA), Alexa Fluor Plus 680-labeled donkey anti-rabbit secondary antibody (1:500, RRID: AB_2762836, Invitrogen, USA). Fluorescence signals were visualized using a Leica DM6B upright digital research microscope (Leica, Germany) or Leica TCS SP8 (Leica, Germany).

**Tracing virus injection**

All viruses in the present study were packaged by BrainVTA (Wuhan, China). The mice were fixed in a stereotactic frame (RWD, Shenzhen, China) under 2% isoflurane anesthesia. A heating pad was used to maintain the body temperature of the mice at 37 °C. Unless otherwise noted, a volume of 100 nl virus was injected per side. The injections were given over 5 min at a rate of 20 nl/min by an infusion pump (Drummond, Alabama, USA) and left in place for 10 min. The stereotaxic coordinates for eLPB are used as following: AP, -5.20 mm, ML, ±1.55 mm and DV, -3.60 mm. The stereotaxic coordinates of CeA are used as following: AP, -0.90 mm; ML, ±2.70 mm; DV, -4.55 mm. Mice were perfused with 0.9% saline, followed by 4% PFA. After 3/4-week transfection, images of the EGFP, mCherry and DsRed signals were visualized to assess the virus-injected positions. Animals with missed injections were excluded from the study.

For anterograde tracing, the *rAAV2/9-ChAT-EGFP* (PT-1722, 2.15E+12 vg/ml) virus was unilaterally injected into the eLPB of WT mice, and the *rAAV2/9-EF1α-DIO-EGFP* (PT-0795, 2.04E+12 vg/ml) were unilaterally injected into the eLPB of ChAT-Cre mice. For retrograde tracing, the *CTB-555* (CTB-02, 1 μg/μl) was unilaterally injected into CeA. In mapping experiment of eLPB^ChAT^-CeA^GABA^ pathway, the *rAAV2/9-ChAT-EGFP* (PT-1722, 2.15E+12 vg/ml) was unilaterally delivered into the eLPB, and the helper viruses that contained *rAAV2/9-VGAT1-CRE* (PT-0346, 3.63E+12 vg/ml, 25 nl), *rAAV2/9-DIO-TVA* (PT-0021, 5.56E+12 vg/ml, 25 nl) and *rAAV2/9-DIO-RVG* (PT-0023, 5.29E+12 vg/ml, 50 nl) were co-injected into the CeA, and followed by the *RV-EnvA-ΔG-DsRed* (R01002, 2.00E+08 IFU/ml) being injected into the CeA 2 weeks later^1^.

**Fluorescent micro-optical sectioning tomography (fMOST)**

In the fMOST, the *rAAV2/9-ChAT-EGFP* (PT-1722, 2.15E+12 vg/ml) virus was unilaterally injected into the eLPB of WT mice. The intact brains were collected and polymerized (45 °C for 2 h, 48 °C for 4 h, 50 °C for 3 h) in a 100% LR white resin and 0.3% Sudan Black B (SBB, Sigma, USA) at a gelatin capsule. The imaging-sectioning cycle was performed using BioMapping 5000N (Oebio, Wuhan, China). The resin-embedded brains were cut and imaged in a 0.01 M PBS bath under a modified fMOST microscope at a voxel resolution of 0.35 × 0.35 × 2 µm^3^. The cutting surface of each 2-µm slice in a coronal plane was imaged, and then removed by a fixed diamond knife. The EGFP channels data were saved at a 16-bit depth in LZW-compression TIFF format. The neuron reconstructions from different datasets were registered to the Allen Institute common coordinate framework version 2 (CCFv2) using BrainsMapi^2^.

**Designer receptor exclusively activated by designer drug (DREADD)**

The chemogenetic activation of eLPB^ChAT^ neurons or projecting terminals from eLPB^ChAT^ neurons using a ChAT-promotor driven virus encoding the neuronal activator DREADD hM3Dq. Adeno-associated virus (AAV) vectors were used to express hM3Dq or control (Go). The Clozapine N-oxide (CNO, 2 mg/kg^3^, HY-17366, MedChemExpress) was used to specifically modulate eLPB^ChAT^ neurons or projecting terminals from eLPB^ChAT^ neurons via interaction with Gq virus 30 min before behavioral tests^4^.

**Patch clamp**

In patch clamp experiment, the *rAAV2/9-ChAT-hM3Dq-mCherry* (PT-2213, 5.54E+12 vg/ml) were bilaterally injected into the eLPBs, followed by the *rAAV2/9-VGAT1-EGFP* (PT-3176, 5.31E+12 vg/ml) being bilaterally delivered into the CeAs of WT mice.

Slices preparation were performed as previously described^5^. Mice were anesthetized with 2% isoflurane and perfused with ice-cold oxygenated modified NMDG artificial cerebrospinal fluid (NMDG ACSF) containing (in mM): 92 N-methyl-d-glucamine (NMDG), 2.5 KCl, 1.2 NaH_2_PO_4_•2H_2_O, 30 NaHCO_3_, 20 HEPES, 25 Glucose, 5 Sodium ascorbate, 2 Thiourea, 3 Sodium Pyruvate, 10 MgSO_4_, 0.5 CaCl_2_•2H_2_O (pH = 7.3-7.4, saturated with 95% O_2_/5% CO_2_). Coronal slices (200 μm) that contained the LPB or CeA were sectioned on a vibrating microtome (VT1000s, Leica, Germany) in ice-cold cutting solution. The brain slices were initially incubated in NMDG ACSF for 9 min at 37 °C, followed by N-2-hydroxyethylpiperazine-N-2-ethanesulfonic acid (HEPES) ACSF cotaining (in mM): 86 NaCl, 2.5 KCl, 1.2 NaH_2_PO_4_•2H_2_O, 35 NaHCO_3_, 20 HEPES, 25 Glucose, 5 Sodium ascorbate, 2 Thiourea, 33 Sodium Pyruvate, 1 MgSO_4_, 2 CaCl_2_•2H_2_O (pH = 7.3-7.4, saturated with 95% O_2_/5% CO_2_). During recordings, the brain slice was continuously perfused with oxygenated ACSF containing (in mM): 119 NaCl, 2.5 KCl, 1 NaH_2_PO_4_•2H_2_O, 26.2 NaHCO_3_, 1.3 MgCl_2_, 2.5 CaCl_2_, 11 D-Glucose (pH = 7.3-7.4, 290-295 mOsm). The slices were recorded while they were bath-perfused (2.5 ml/min) at 30°C by an in-line solution heater (TC-324C, Warner Instruments, USA). The internal solution that contained (in mM) 130 K Gluconate, 7 KCl, 0.4 EGTA, 4 Mg-ATP, 0.4 Na-GTP, 10 HEPES, 1 L-glutathione, 7.5 Sodium creatine phosphate dibasic tetrahydrate (pH = 7.3-7.4, 290 mOsm) was used in the current-clamp recordings, while internal solution that contained (in mM) 130 CsMeSO_4_, 10 NaCl, 10 EGTA, 4 Mg-ATP, 0.3 Na-GTP, 10 HEPES (pH = 7.3-7.4, 290 mOsm) was used in the voltage-clamp recordings. The signals were acquired with a Multiclamp 700B amplifier (Molecular Devices, USA), low-pass filtered at 4.0 kHz, digitized at 10 kHz and analyzed with Clampfit 10.5 software (Molecular Devices, USA).

The spontaneous action potentials (sAP) were recorded under current-clamp mode in mCherry-positive neurons (red) in the eLPB using an Olympus BX51WIF microscope optical system combined with a Digital Camera (C11440-42U, Hamamatsu, Japan). To activate eLPB^ChAT^ neurons, 10 μM CNO was added in the recording ACSF solution.

The spontaneous EPSC (sEPSC) were performed under voltage-clamp (Voltage holding at −70mV) mode^6^ in EGFP-positive GABA neurons (green) in the CeA. To activate the terminals of eLPB^ChAT^ neurons within the CeA, 10 μM CNO^4^ were added in the recording ACSF solution. During patch clamp recording, the ACSF solution with the addition of 50 µM picrotoxin^7^ to block GABA_A_ receptors. The 5 μM mecamylamine (MEC)^8^ were used to non-specifically inhibit nicotinic acetylcholine receptors (nAChRs) on the CeA^GABA^ neurons.

**Fiber photometry**

In the fiber photometry experiment, the *rAAV2/9-VGAT1-GCaMp6m* (PT-3317) virus was bilaterally injected into CeAs, and the *rAAV2/9-ChAT-hM3Dq* (Gq, PT-2874, 5.54E+12 vg/ml) or *rAAV2/9-ChAT* (Go, PT-0607, 5.50E+12 vg/ml) virus was bilaterally injected into the eLPBs of WT mice. An optical fiber (200 μm outer diameter, 0.37 numerical aperture, Inper Ltd., China) was placed 150 μm above the viral injection site. Fiber photometry experiments were performed as previously described^9^. The calcium-dependent fluorescence signals were obtained by stimulating cells that transfected *GCaMp6m* virus with a 470 nm LED (35-40 μW at fiber tip), while calcium-independent signals were obtained by stimulating these cells with a 405 nm LED (15-20 μW at fiber tip). The LED lights of 470 nm and 405 nm were alternated at 66 fps and light emission was recorded using sCMOS camera containing the entire fiber bundle (2 m in length, NA = 0.37, 200 μm core, Inper Ltd.). The analog voltage signals fluorescent was filtered at 30 Hz and digitalized at 100 Hz. The GCaMp6m signals were recorded and analyzed by Inper Studio Multi Color EVAL15 software (Inper Ltd., China) and Inper Data Process V0.5.9 (Inper Ltd., China), respectively. F0 is the baseline fluorescence signal which was recorded for 1 min prior to CNO treatment. F is the real-time fluorescence signal which was recorded at 0 min, 10 min, 20 min, 30 min, 35 min, 40 min, 45 min and 50 min after CNO treatment. Each recording trail was with 1 min recording duration and 4 min interval. The values of ΔF/F are calculated by (F-F0)/F0. The area under curve (AUC) is the integral under recording duration related to corresponding baseline at every trial.

**Conditioned place preference (CPP)**

In CPP experiment, two cohorts of WT mice were exposed to virus injections. In cohort-1 mice, the *rAAV2/9-ChAT-hM3Dq-mCherry* (Gq, PT-2213, 5.54E+12 vg/ml) or *rAAV2/9-ChAT* (Go, PT-0607, 5.50E+12 vg/ml) was bilaterally injected into the eLPBs, forming eLPB-Gq mice and eLPB-Go mice. In cohort-2 mice, the *rAAV2/9-ChAT-DIO-hM3Dq-mCherry* (Gq, PT-2825, 5.08E+12 vg/ml) or *rAAV2/9-ChAT* (Go, PT-0607, 5.50E+12 vg/ml) were bilaterally delivered into the eLPBs, followed by the rAAV2/retro-Cre-EGFP (PT-1168, 5.25E+12 vg/ml, 150 nl) being bilaterally injected into the CeAs, forming CeA-Gq mice and CeA-Go mice. Mice received CNO (2 mg/kg, i.p.）30 min before each behavioral test.

METH CPP procedures were performed in the TopScan3D CPP apparatus (CleverSys, VA, USA), which is constructed of two distinct chambers (30.5 × 25 × 32 cm each) separated by a removable guillotine door. The CPP procedure^10,11^ consisted of five phases: the pre-conditioning test (Pre-test, day 0), conditioning (CPP training, days 1-7), post-conditioning test (Test, day 8), extinction training (Extinction, day 9-23), and METH priming-induced reinstatement test (Priming, day 24). Baseline preference was assessed by placing the mice in a random chamber of the CPP apparatus and allowing them to explore all two chambers freely for 15 min. Conditioning was confined to preferred chamber for 45 min paired with a saline (0.2 ml, i.p.) injection in the morning, and non- preferred chamber for 45 min paired with a METH (3 mg/kg, i.p.) injection in the afternoon for seven consecutive days. During the Test and Extinction, mice allowed to freely access the two chambers without any drug treatment for 15 min or 45 min, respectively. For the METH priming-induced reinstatement test, mice were injected with METH (0.5 mg/kg, i.p.) and then allowed to freely explore both chambers for 15 min.

The CPP score was calculated by subtracting the duration spent in saline-paired chamber from the METH-paired chamber, and the ΔCPP score was the priming CPP score minus extinction CPP score.

**Statistical analysis**

Statistical analysis was carried out using GraphPad Prism 8.0 software. For electrophysiology experiments, data are presented as the mean ± SEM. Other data are presented as the mean ± SD. The data of sAP and CPP scores were analyzed with paired t-tests. The data of sEPSC were analyzed by repeated measures of one-way ANOVA with Tukey’s post hoc tests. The data of fiber photometry were analyzed by repeated measures of two-way ANOVA with Sidak post hoc tests. The data of ΔCPP scores were analyzed by unpaired t-tests. Statistical significance was set as *P* < 0.05.

**References**

1. Zhou W, Jin Y, Meng Q*, et al*. A neural circuit for comorbid depressive symptoms in chronic pain. *Nat Neurosci*. Oct 2019;22(10):1649-1658. doi:10.1038/s41593-019-0468-2

2. Lin R, Liang J, Wang R*, et al*. The Raphe Dopamine System Controls the Expression of Incentive Memory. *Neuron*. Jun 2 2021;109(11):1906. doi:10.1016/j.neuron.2021.05.011

3. Yuan F, Jiang H, Yin H*, et al*. Activation of GCN2/ATF4 signals in amygdalar PKC-delta neurons promotes WAT browning under leucine deprivation. *Nat Commun*. Jun 5 2020;11(1):2847. doi:10.1038/s41467-020-16662-2

4. Kayyal H, Yiannakas A, Kolatt Chandran S, Khamaisy M, Sharma V, Rosenblum K. Activity of Insula to Basolateral Amygdala Projecting Neurons is Necessary and Sufficient for Taste Valence Representation. *J Neurosci*. Nov 20 2019;39(47):9369-9382. doi:10.1523/JNEUROSCI.0752-19.2019

5. Ge F, Mu P, Guo R*, et al*. Chronic sleep fragmentation enhances habenula cholinergic neural activity. *Mol Psychiatry*. Mar 2021;26(3):941-954. doi:10.1038/s41380-019-0419-z

6. Park S, Williams KW, Liu C, Sohn JW. A neural basis for tonic suppression of sodium appetite. *Nat Neurosci*. Mar 2020;23(3):423-432. doi:10.1038/s41593-019-0573-2

7. Venniro M, Caprioli D, Zhang M*, et al*. The Anterior Insular Cortex-->Central Amygdala Glutamatergic Pathway Is Critical to Relapse after Contingency Management. *Neuron*. Oct 11 2017;96(2):414-427 e8. doi:10.1016/j.neuron.2017.09.024

8. Lucas-Meunier E, Monier C, Amar M, Baux G, Fregnac Y, Fossier P. Involvement of nicotinic and muscarinic receptors in the endogenous cholinergic modulation of the balance between excitation and inhibition in the young rat visual cortex. *Cereb Cortex*. Oct 2009;19(10):2411-27. doi:10.1093/cercor/bhn258

9. Grund T, Tang Y, Benusiglio D*, et al*. Chemogenetic activation of oxytocin neurons: Temporal dynamics, hormonal release, and behavioral consequences. *Psychoneuroendocrinology*. Aug 2019;106:77-84. doi:10.1016/j.psyneuen.2019.03.019

10. Karimi-Haghighi S, Haghparast A. Cannabidiol inhibits priming-induced reinstatement of methamphetamine in REM sleep deprived rats. *Prog Neuropsychopharmacol Biol Psychiatry*. Mar 2 2018;82:307-313. doi:10.1016/j.pnpbp.2017.08.022

11. Shaham Y, Shalev U, Lu L, de Wit H, Stewart J. The reinstatement model of drug relapse: history, methodology and major findings. *Psychopharmacology (Berl)*. Jul 2003;168(1-2):3-20. doi:10.1007/s00213-002-1224-x
