# Supplementary material for: A novel cholinergic projection from the lateral parabrachial nucleus and its role in methamphetamine-primed conditioned place preference
Source: Brain Commun. 2022 Aug 30;4(5):fcac219. doi: 10.1093/braincomms/fcac219 (PMC9536296; doi:10.1093/braincomms/fcac219)

Supplementary Figure legends

Supplementary Figure 1. Anatomical dissection of eLPB ^{ChAT}-CeA^{GABA} pathway. (A) The three-dimensional reconstructions of LPB^{ChAT} projections in the brain. The sagittal (left) and top (right) representative images of *ChAT* virus labeled projections from the LPB in WT mice. Scale bar, 50 pixels. **(B)** Schematic diagram of the *rAAV2/9-EF1α-DIO-EGFP* injection in ChAT-Cre mice. **(C)** Representative images of EGFP-labled virus and ChAT-positive neurons in the eLPB of ChAT-Cre mice. Scale bar, 400 μm. **(D)** Representative images of EGFP-labeled viral expression within the CeA. Scale bar, 400 μm. **(E)** Schematic diagram of *CTB-555* injection in WT mice. **(F)** Representative images of *CTB-555* infusion in the CeA. Scale bar, 400 μm. **(G)** *CTB-555*-transfected and ChAT-positive neurons in the eLPB. Scale bar, 400 μm. **(H)** The percentage of *CTB-555*-transfected cells out of ChAT-positive neurons, and ChAT-positive neurons out of *CTB-555*-transfected cells in the eLPB, n = 3 mice.

Supplementary Figure 2. Physiological innervation of eLPB^{ChAT}-CeA^{GABA} pathway. (A) The proportions of neurons co-labeled with GCaMp6m and GAD67 antibody. Scale bar, 200 μm. **(B)** The percentage of GCaMp6m^{+}, GAD67^{+} cells relative to GAD67^{+} (top) or GCaMp6m^{+} (bottom, all the GCaMp6m^{+} cells 100% co-labeling with GAD67 antibody) cells in the CeA. n = 3 mice. **(C)** The representative images of *GCaMp6m*-labeled virus and c-Fos-positive neurons in the CeA of WT mice. Scale bar, 200 μm. **(D)** The percentage of c-Fos-positive cells out of *GCaMp6m*-positive neurons in the CeA. n = 6 mice.

Supplementary Figure 3. The role of eLPB^{ChAT}-CeA^{GABA} pathway in METH-primed CPP. (A) The proportions of neurons co-labeled with mCherry and ChAT antibody in the LPB. Scale bar, 100 μm. **(B)** The percentage of mCherry^{+}, ChAT^{+} cells relative to ChAT^{+} (right) or mCherry^{+} (right) cells in the LPB, n = 5 mice. **(C)** CPP scores in eLPB-Go and eLPB-Gq mice during baseline and test (eLPB-Go, n = 8 mice. eLPB-Gq, n = 10 mice. Two-way ANOVA with *Sidak post hoc* tests). **(D)** CPP scores in eLPB-Go and eLPB-Gq mice during extinction (eLPB-Go, n = 8 mice. eLPB-Gq, n = 10 mice. Two-way ANOVA with *Sidak post hoc* tests). **(E)** The total distance traveled by eLPB-Go and eLPB-Gq mice during METH-primed CPP (eLPB-Go, n = 8 mice. eLPB-Gq, n = 10 mice.

Unpaired *t*-tests). **(F)** CPP scores in CeA-Go and CeA-Gq mice during baseline and test CeA-Go, n = 6 mice. CeA-Gq, n = 6 mice. Two-way ANOVA with *Sidak post hoc* tests). **(G)** CPP scores in CeA-Go and CeA-Gq mice during extinction (CeA-Go, n = 6 mice. CeA-Gq, n = 6 mice. Two-way ANOVA with *Sidak post hoc tests*). **(H)** The total distance traveled by CeA-Go and CeA-Gq mice during METH-primed CPP (CeA-Go, n = 6 mice. CeA-Gq, n = 6 mice. Unpaired *t*-tests).

Supplementary Figure 1

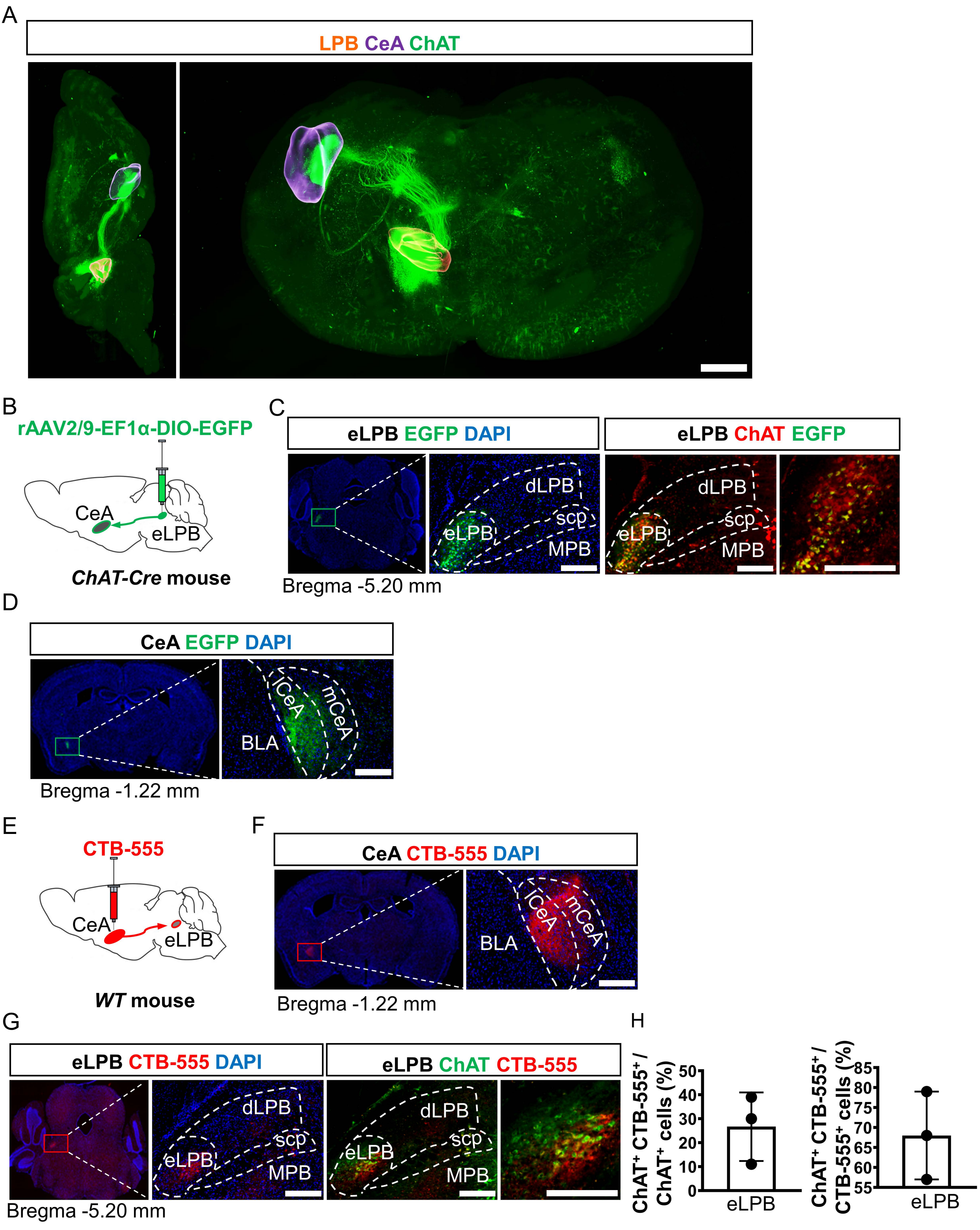

Supplementary Figure 2

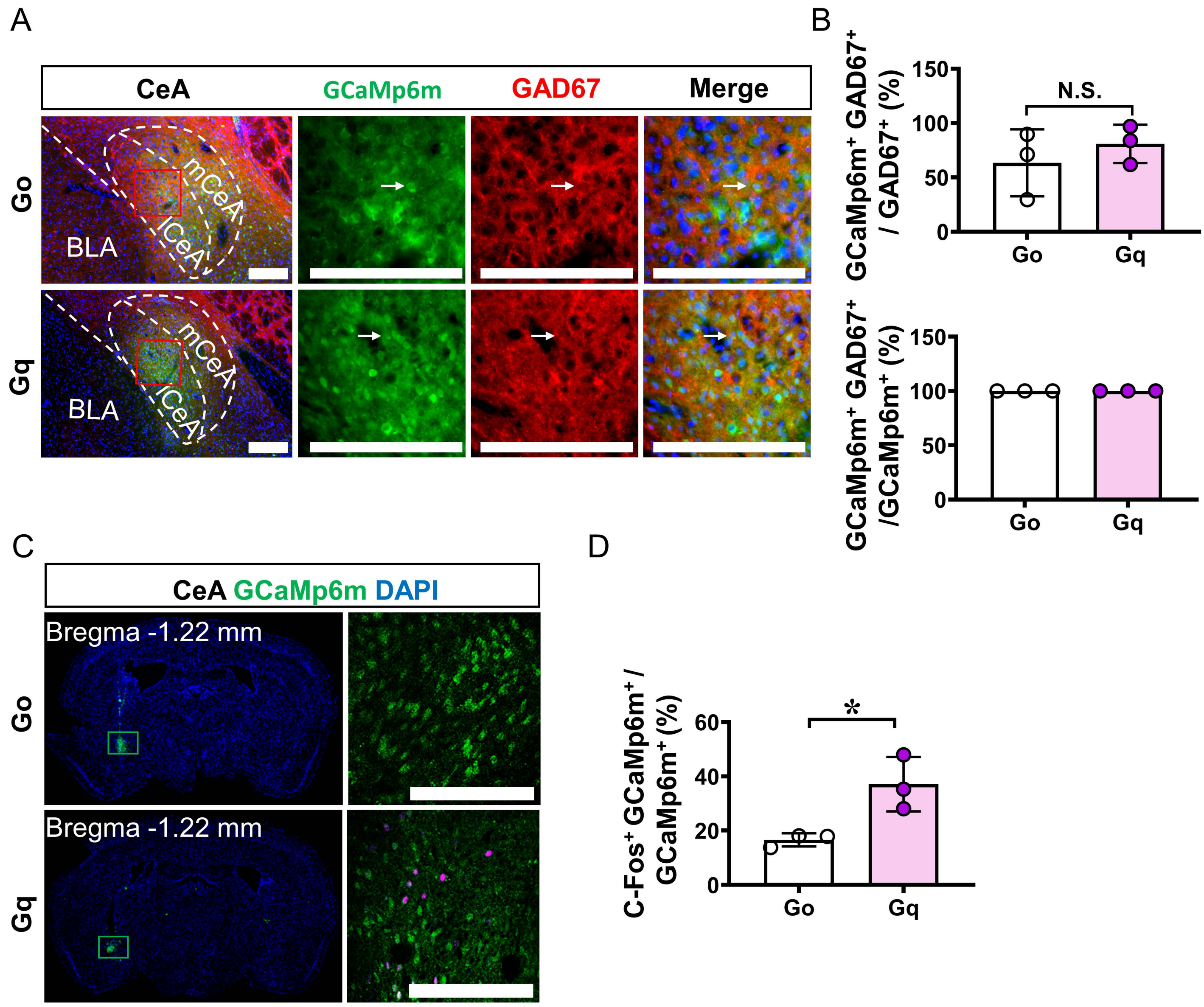

Supplementary Figure 3

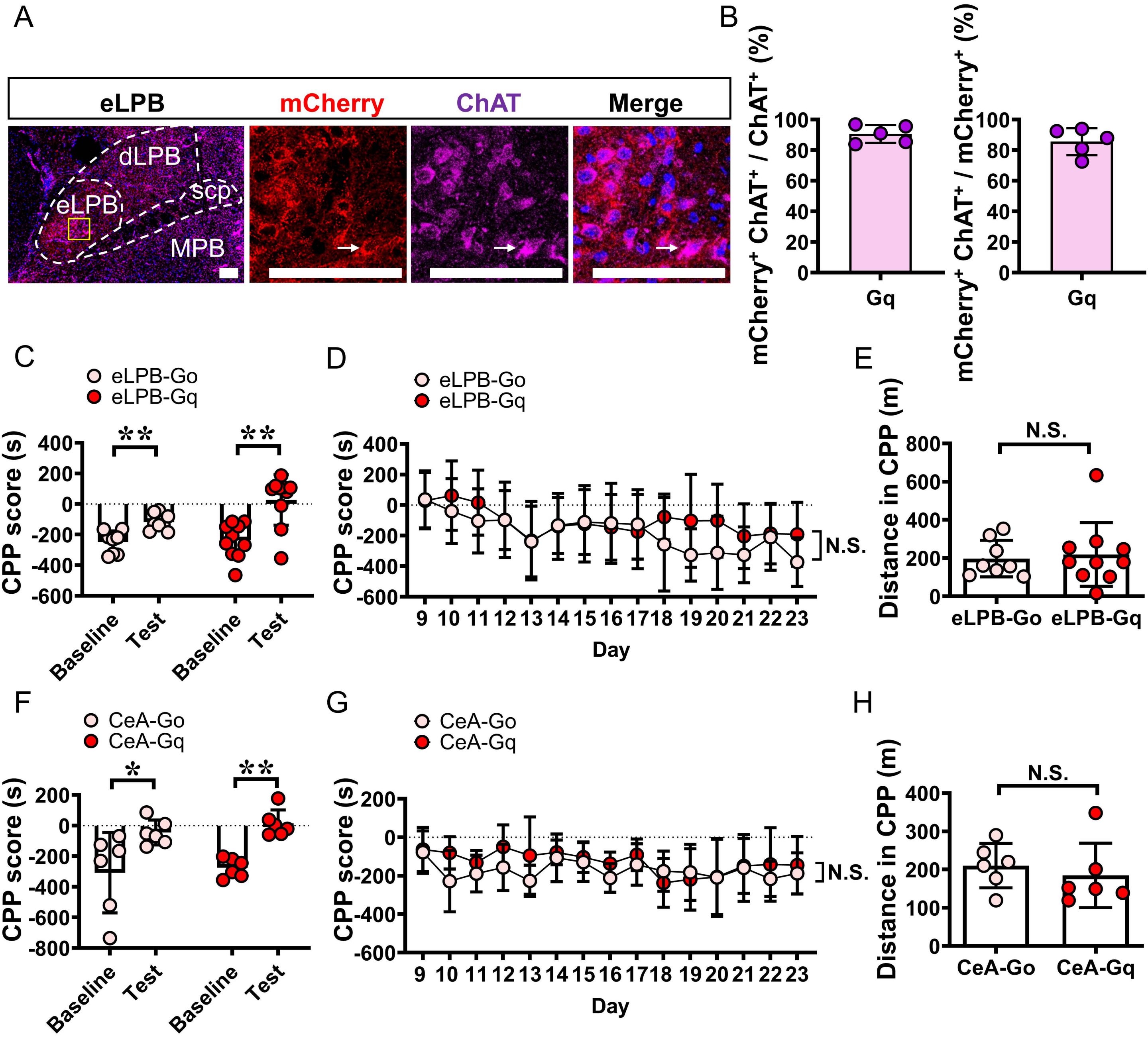

Supplement: fcac219_Supplementary_Data [file fcac219_Supplementary_Data.zip › Supplementary data.pdf]
